# Human Alpha Galactosidases Transiently Produced in *Nicotiana benthamiana* Leaves: New Insights in Substrate Specificities with Relevance for Fabry Disease

**DOI:** 10.3389/fpls.2017.01026

**Published:** 2017-06-21

**Authors:** Kassiani Kytidou, Thomas J. M. Beenakker, Lotte B. Westerhof, Cornelis H. Hokke, Geri F. Moolenaar, Nora Goosen, Mina Mirzaian, Maria J. Ferraz, Mark de Geus, Wouter W. Kallemeijn, Herman S. Overkleeft, Rolf G. Boot, Arjen Schots, Dirk Bosch, Johannes M. F. G. Aerts

**Affiliations:** ^1^Department of Medical Biochemistry, Leiden Institute of ChemistryLeiden, Netherlands; ^2^Department of Bio-organic Synthesis, Leiden Institute of ChemistryLeiden, Netherlands; ^3^Wageningen University and Research, Plant Sciences GroupWageningen, Netherlands; ^4^Department of Parasitology, Centre of Infectious Diseases, Leiden University Medical CenterLeiden, Netherlands; ^5^Cloning and Protein Purification Facility of Leiden Institute of ChemistryLeiden, Netherlands

**Keywords:** α-galactosidase, α-*N*-acetyl-galactosaminidase, Fabry disease, therapy, recombinant enzyme, *Nicotiana benthamiana*

## Abstract

Deficiency of α-galactosidase A (α-GAL) causes Fabry disease (FD), an X-linked storage disease of the glycosphingolipid globtriaosylcerammide (Gb3) in lysosomes of various cells and elevated plasma globotriaosylsphingosine (Lyso-Gb3) toxic for podocytes and nociceptive neurons. Enzyme replacement therapy is used to treat the disease, but clinical efficacy is limited in many male FD patients due to development of neutralizing antibodies (Ab). Therapeutic use of modified lysosomal α-*N*-acetyl-galactosaminidase (α-NAGAL) with increased α-galactosidase activity (α-NAGAL^EL^) has therefore been suggested. We transiently produced in *Nicotiana benthamiana* leaves functional α-GAL, α-NAGAL, and α-NAGAL^EL^ enzymes for research purposes. All enzymes could be visualized with activity-based probes covalently binding in their catalytic pocket. Characterization of purified proteins indicated that α-NAGAL^EL^ is improved in activity toward artificial 4MU-α-galactopyranoside. Recombinant α-NAGAL^EL^ and α-NAGAL are not neutralized by Ab-positive FD serum tested and are more stable in human plasma than α-GAL. Both enzymes hydrolyze the lipid substrates Gb3 and Lyso-Gb3 accumulating in Fabry patients. The addition to FD sera of α-NAGAL^EL^, and to a lesser extent that of α-NAGAL, results in a reduction of the toxic Lyso-Gb3. In conclusion, our study suggests that modified α-NAGAL^EL^ might reduce excessive Lyso-Gb3 in FD serum. This neo-enzyme can be produced in *Nicotiana benthamiana* and might be further developed for the treatment of FD aiming at reduction of circulating Lyso-Gb3.

## Introduction

Deficiencies in lysosomal enzymes are the cause of various inherited lysosomal storage disorders in humans (Futerman and Van Meer, 2004). Examples are Gaucher disease (GD), Pompe disease and Fabry disease (FD) with deficiencies of acid beta-glucosidase (glucocerebrosidase, GBA), acid alpha-glucosidase and acid alpha-galactosidase (α-GAL) respectively. For each of these diseases, enzyme replacement therapy (ERT) approaches have been designed and are applied with variable success. Highly efficient is ERT for GD resulting in reversal and/or prevention of organomegaly and hematological abnormalities in non-neuropathic type 1 patients (Barton et al., 1990). ERT of Pompe disease has been shown to increase the life expectancy in patients suffering from the infantile form of the disorder (Van den Hout et al., 2000). Two similar approaches were also developed for FD using human α-GAL produced in Chinese hamster ovary cells (agalsidase beta; Fabrazyme^TM^; Sanofi-Genzyme) or human fibroblasts (agalsidase alfa; Replagal^TM^; Shire) (Eng et al., 2001; Schiffmann et al., 2001). Both enzyme preparations were registered in August 2001 as orphan drug in Europe, but only Fabrazyme was approved by the FDA in the United States (Desnick, 2004). Fabrazyme and Replagal are infused bi-weekly at a dose of 1.0 and 0.2 mg/kg body weight, respectively (Eng et al., 2001; Schiffmann et al., 2001).

α-Galactosidase A (α-GAL; E.C. 3.2.1.22) is encoded by the *GLA* (ID: 2717) gene at locus Xq22 (Desnick et al., 2001). The enzyme is synthesized as 429 amino acid precursor from which the signal peptide is removed to yield a 398 amino acid glycoprotein forming a homodimer (Brady et al., 1967; Hamers et al., 1977; Bishop et al., 1988; Desnick et al., 2001). Formation of mannose-6-moieties (M6P) in the 3 *N-*linked glycans of α-GAL allows transport to lysosomes by mannose-6-phosphate receptors (M6PR) (Sakuraba et al., 2005). The natural substrates of α-GAL are glycosphingolipids with terminal α-galactosyl moieties, including globotriaosylceramide (Gb3; ceramidetrihexoside: CTH), galabiosylceramide, and blood group B, B1, and P_1_ antigens (Sweeley and Klionsky, 1963; Desnick et al., 1996). In various cell types of classic male FD patients Gb3 accumulates in intra-lysosomal lipid deposits. Both Fabrazyme and Replagal are aimed to be delivered to lysosomes of cells by M6PR mediated endocytosis (Sakuraba et al., 2005). Unfortunately, the evaluation of long-term ERT of FD patients indicates that clinical efficacy is relatively poor (Schuller et al., 2015). ERT treatments result in the case of some FD patients in stabilization of disease, but progression of symptoms has also been observed (Blom et al., 2003; Lin et al., 2009). The poor response to ERT of classic FD males, usually completely lacking α-GAL protein, is partially explained by the common induction in these individuals of neutralizing antibodies (Ab) against the therapeutic enzyme (Linthorst et al., 2004).

A recent important insight on pathogenesis of FD stems from the notion that the storage lipid Gb3 is partly actively converted to its sphingoid base globotriaosylsphingosine (Lyso-Gb3) in lysosomes (Aerts et al., 2008; Ferraz et al., 2016a). In symptomatic FD male plasma Lyso-Gb3 is chronically several 100-fold increased, and symptomatic female FD patients also show increased levels of the sphingoid base (Gold et al., 2013; Ferraz et al., 2016a). Excessive circulating Lyso-Gb3 seems a culprit, contributing to renal complications and neuropathic pain in FD patients as the results of toxicity for podocytes and nociceptive neurons respectively (Choi et al., 2015; Sanchez-Niño et al., 2015). In line with this, FD patients with Abs show a relapse of plasma Lyso-Gb3 to pre-treatment values (Ohashi et al., 2007; van Breemen et al., 2011; Rombach et al., 2012). In view of the toxicity of Lyso-Gb3 a treatment based on enzyme supplementation aiming to specifically reduce this lipid might be considered. Ideally, such therapeutic enzyme should not induce neutralizing antibodies in FD males as occurs with Fabrazyme and Replagal.

Plants, among others *Nicotiana benthamiana*(N. benthamiana), have been shown to be excellent production platforms of therapeutic enzymes (Fischer et al., 2004; Gomord and Faye, 2004). Besides the amply reviewed advantages such as associated low costs, feasibility of large scale production and reduced risk for contaminating animal viruses and prions, plants may be engineered to process recombinant proteins with all post-translational modifications required for desired bioactivity and pharmacokinetics. Shaaltiel et al. (2007) were among the first to develop the production of a lysosomal hydrolase in plant cells for therapeutic use in humans. They generated recombinant glucocerebrosidase in carrot cells for the treatment of type 1 GD. Clinical investigations with GD patients demonstrated that the therapeutic efficacy of the plant-produced enzyme preparation is on *a par* with recombinant enzyme conventionally produced in mammalian cells. Moreover, no significant immune responses to the plant-produced glycoprotein as such were noted. These findings promoted swift registration of taliglucerase (Uplyso^TM^; Protalix) as drug for type 1 GD in Europe, Israel, and the United States (Van Dussen et al., 2013). Another recent example forms the production in tobacco of acid alpha glucosidase for treatment of Pompe disease (Su et al., 2015). Furthermore, in *Nicotiana tabacum* cells a PEGylated human α-GAL enzyme has been produced for treatment of FD (Kizhner et al., 2014). The same enzyme was also recombinantly produced in an engineered moss cell line by Shen et al. (2016) and shown to undergo mannose receptor mediated uptake.

The unfortunate detrimental immune response to infused human α-GAL’s in most FD males, leading to neutralizing antibodies, inspired Sakubara and colleagues to propose the use of a modified enzyme (Tajima et al., 2009). Their alternative approach elegantly exploits the existence of a homologous lysosomal enzyme named α-galactosidase B or α-*N*-acetyl-galactosaminidase (α-NAGAL). It is encoded by the *NAGA* gene (ID: 4668) (22q13.2) arisen by gene duplication of the *GLA* gene. Until the late 70s both enzymes were actually considered as different isoforms of the same protein (Schram et al., 1977). Mature α-NAGAL, a 411 amino acid glycoprotein with 4 *N-*linked glycans still shows considerably structural similarity to α-GAL. The enzyme α-NAGAL exerts α-*N*-acetyl-galactosaminidase activity, but also hydrolyses at low rate artificial α-galactosides like 4-methylumbelliferyl-α-galactopyranoside (4MU-α-GAL) or *p*-nitrophenyl-α-galactopyranoside (pNP-α-GAL). The similarity of α-GAL and α-NAGAL is also revealed by recently developed activity-based probes (ABPs) that bind covalently to the catalytic nucleophile of both retaining glycosidases (Willems et al., 2014). In the present study, we used a similar alpha-galactosyl configured cyclophellitol-aziridine ABP equipped with a Cy5 fluorophore, again labeling both α-GAL and α-NAGAL. X-ray crystallography of α-GAL and α-NAGAL provided a structural basis for the specificity of both proteins for substrates (Tomasic et al., 2010). The introduction of two amino acid substitutions in the catalytic pocket of α-NAGAL, Ser188Glu and Ala191Leu, suffices to increase about 40 times the ability of the neo-enzyme (α-NAGAL^EL^) to hydrolyse 4MU-α-GAL (Tajima et al., 2009). Furthermore, it was described that α-NAGAL^EL^ reduces Gb3 in cultured fibroblasts from a FD patient. Intravenous administration of α-NAGAL^EL^ in FD mice partially reduced Gb3 storage in liver, kidney, and heart (Tajima et al., 2009). Garman recapitulated some of the findings by demonstration of about 4.5-fold increased activity of α-NAGAL^EL^ toward *p*-Nitrophenyl-α-D-galactoside, PNP-α-GAL (Tomasic et al., 2010). The enzymes α-GAL and α-NAGAL differ not only in affinity for the sugar-moiety of natural substrates, but also their aglycon moieties (Clark and Garman, 2009; Guce et al., 2010). Whereas α-GAL degrades glycosphingolipids, α-NAGAL degrades glycopeptides and oligosaccharides as indicated by storage materials in Schindler disease (inherited α-NAGAL deficiency).

In our present study, we investigated whether it is feasible to produce in *N. benthamiana* leaves α-GAL, α-NAGAL, and α-NAGAL^EL^. All plant-produced enzymes were active and could be labeled by the Cy5 equipped ABP. The glycosidases were purified to homogeneity and characterized regarding enzymatic activity toward artificial alpha-galactoside and alpha-*N*-acetylkgalactosiminide substrates, and their *N-*glycan composition. In addition, we produced the same enzymes in HEK293 cells, showing no difference in specific activities to the ones produced in the *N. benthamiana* leaves. Moreover, we established whether the enzymes cross react with antibodies in serum of an FD patient with neutralizing activity (Linthorst et al., 2004). Next, we studied the activity of the various enzymes toward lipid substrates, in particular Gb3 and Lyso-Gb3. Finally, we examined the stability of various enzymes in plasma and their ability to degrade excessive Lyso-Gb3 in FD sera using LC–MS/MS and isotope-encoded internal standards (Gold et al., 2013; Ferraz et al., 2016a). From the results obtained we conclude that it seems feasible to produce a modified α-NAGAL^EL^ that is more stable in human plasma than α-GAL and is better able to degrade excessive Lyso-Gb3 in FD serum. Further tailoring of an enzyme to optimally degrade circulating Lyso-Gb3 in the blood of FD patients is a therapeutic avenue to be considered, and plants might be used as a protein production platform for this purpose.

## Materials and Methods

### Plants

*Nicotiana benthamiana* plants were grown at 21°C and 60–70% humidity in the Unifarm greenhouses of Wageningen University (Westerhof et al., 2014).

### Chemicals

All chemicals were obtained from Sigma (Germany) if not indicated otherwise. Fluorescent NBD-lipids and pure lipids were purchased from Avanti (Alabama, United States). Antibodies purchased from Abcam (Cambridge, MA, United States).

### Activity Based Probe (ABP)

The fluorescent ABP directed against α-galactosidases was produced based on previously described synthesis (Willems et al., 2014). Alpha-Galactopyranose configured cyclophellitol-aziridine was grafted with Cy5 as fluorophore, as described in detail in the Supplementary Material (Scheme 1). The newly synthesized probe was examined on reactivity with human recombinant α-GAL (Fabrazyme, Sanofi-Genzyme, Cambridge, MA, United States) (Supplementary Figures S1A,B).

### Preparation of Human α-GAL, α-NAGAL, and α-NAGAL^EL^ (S188E; A191L) Plant Expression Vectors

Both *GLA* (ID: 2717) and *NAGA* (ID: 4668) were amplified from MegaMan Human Transcriptome cDNA library (Stratagene). The coding region of genes was amplified by Phusion^®^ HighFidelity PCR MasterMix (BioLabs) using the following primers: *GLA* sense: 5′-CTCATGAGTGCCAAGACCAACCTCTTCCTCTTCCTCATCTTCTCCCTCCTGCTCTCCCTC TCCTCCGCCCTGGACAATGGATTGGCAAG-3′, *GLA*antisense: 5′-CCCGTACGTTAAAG TAAGTCT-3′, *NAGA* sense: 5′-CTCATGAGTGCCAAGACCAACCTCTTCCTCTTCCTCATC TTCTCCCTCCTGCTCTCCCTCTCCTCCGCC-3′, *NAGA* antisense: 5′-CCGTACGTCACTGCT GGGACA-3′. The genes were amplified between BspHI and BsiWI restriction sites, lacking the sequence encoding for their native signal peptide. Instead, the native signal peptide of *Arabidopsis thaliana* chitinase was added at primer sequences. Insertion of mutations S188E and A191L in *NAGA* gene was achieved via overlap extension PCR. PCR reactions were performed using the following primers: *NAGA* mutant antisense: 5′-CCTTCATAGAGTGGCCACTCGCAGGAG AAGGC-3′, *NAGA* mutant sense: 5′-GCCTTCTCCTGCGAGTGGCCACTCTATGAAGG-3′. The elements were cloned into pGEM -T Easy Vector System (Promega), following electroporation of DH5α *Escherichia coli* cells. After clone selection and confirmation of sequences by sequencing (Macrogen), the complete open reading frames (ORFs) of *GLA, NAGA*, and *NAGA^EL^* were inserted in plant expression vectors. A modified version of pMDC32 expression vector, named pHYG (Westerhof et al., 2012), was used as the main plant expression vector during all experiments. The vector was digested with Acc65I and NcoI, leaving compatible overhangs for ligation of the elements flanked between BspHI and BsiWI restriction sites. All constructs were under the control of cauliflower mosaic virus 35S constitutive promoter, with duplicate enhancer (d35S) and the nopaline synthase terminator (Tnos) derived from *Agrobacterium tumefaciens*. The pHYG vectors harboring the genes were used for transformation of *Agrobacterium tumefaciens* strain MOG101, following *N. benthamiana* plant leaf infiltrations.

### *Agrobacterium tumefaciens* Transient Transformation Assay

*Agrobacterium tumefaciens* cultures were grown as previously described (Westerhof et al., 2012, 2014). The constructs were co-expressed or not with the tomato bushy stunt virus silencing inhibitor p19 (Voinnet et al., 2003), by mixing the bacterial cultures 1:1 following 1–2 h incubation at room temperature.

### Infiltration of Plants, Harvesting, and Lysate Preparation

The inoculated bacterial cultures were used for infiltration of the two youngest leaves of 5–6 weeks old *N. benthamiana* plants, to ensure optimum protein expression, as previously described (Westerhof et al., 2014). Whole leaves and leaf disks (50 mm) were harvested at different days post-infiltration (dpi), snap-frozen and homogenized in liquid nitrogen. Homogenization was performed with a tissue lyser (AKA Qiagen TissueLyser II) at 30 rounds/sec for 1 min. Extraction of total soluble proteins of samples was achieved using 2–3 ml per leaf gram of extraction buffer (30 mM citrate-phosphate buffer, pH 6.3, containing 2% (w/v) polyvinylpolypyrrolidone, 0.1% (v/v) Tween 20, 0.5 M NaCl, and protease inhibitor by Roche, EDTA free) following homogenization at same tissue lyser program. When whole leaves were used, homogenization was performed by grinding with a mortar and pestle. Samples were next centrifuged for 15 min at 15000 *g* (F13S 14 × 50 cy, Sorvall rotor), at 4°C. The supernatant was collected and used for further analysis or it was stored at -80°C after desalting at G25-Sephadex column.

### Enzyme Purification

As first step in the purification of all enzymes, a 5 ml column of Concanavalin-A-Sepharose (GE healthcare Bio-Sciences) was used. The column was equilibrated with 40 ml washing buffer (0.1 M sodium acetate, 0.1 M NaCl, 1 mM MgCl_2_, 1 mM CaCl_2_, 1 mM MnCl_2_, pH 6.0). Then, protein sample (lysate) was applied to the column, 1:1 diluted in washing buffer (0.5 ml/min loading conditions). For α-GAL 49 ml of protein lysate was applied on the column, for α-NAGAL 38 ml and α-NAGAL^EL^ 25 ml. Proteins were eluted with 30 ml elution buffer (washing buffer supplemented with 0.9 M methyl-α-mannoside and 0.9 M methyl-α-glucoside). Ten fractions of 1 ml showing highest levels of enzymatic activity were pooled (post-ConA). Next, for α-GAL purification, 10 ml of post-ConA was loaded on an ultrafiltration device (Centricon Plus-20, 15 ml with 10 kDa molecular cutoff, Millipore, Bedford, MA, United States) to remove methyl-monosaccharides and buffer exchanged to 20 mM sodium acetate, pH 5.0, until obtaining a volume of 3 ml. Dialysis of the sample was continued in 20 mM sodium acetate buffer, pH 5.0, 4°C, overnight. A HiTrap SP HP column (1 ml; GE healthcare Bio-Sciences) was equilibrated with 15 ml binding buffer: 20 mM sodium acetate buffer, pH 5.0. The sample, 3 ml, was applied on the column which was extensively washed with 10 ml binding buffer afterward. Then, protein was eluted using a gradient of 0–300 mM NaCl, 15 ml. Enzyme eluted in 0.5 ml fractions 2–4, which were pooled. For wild type α-NAGAL and modified α-NAGAL^EL^, post-ConA, 2–3 ml, was buffer exchanged via ultrafiltration to 10 mM potassium phosphate buffer, KPi (pH 6.3) with 10 mM NaCl. Next, sample was dialyzed in the same buffer to further remove any methyl-monosaccharides. After this, sample was applied on a HiTrap Q HP column (1 ml; GE healthcare Bio-Sciences). Anion exchange chromatography was performed using a gradient of 10–600 mM NaCl in 10 mM KPi (pH 6.3). Enzymes eluted in 0.5 ml fractions 2–4, which were pooled. As final purification step for all enzymes gel filtration was used. A Superdex^TM^ 200 Increase 10/300 GL (GE healthcare Bio-Sciences) column was prior equilibrated with 50 ml of 20 mM sodium acetate buffer with 150 mM NaCl, pH 5.0. Samples were applied and chromatography performed at a flow 0.75 ml/min. 0.2 ml fractions were collected and analyzed on enzymatic activity. The final material was snap frozen in liquid nitrogen and stored at -80°C until further use.

### Overproduction of α-GAL, α-NAGAL, and α-NAGAL^EL^ in HEK293 Cells

Vectors for human cell line HEK293 were produced as follows: the complete OPFs of human α-GAL, α-NAGAL, and α-NAGAL^EL^ were amplified from previous pGEMt easy vectors harboring the genes with their native signal peptide, via PFX50 high fidelity DNA polymerase (Invitrogen), using the primers below. *GLA* sense: 5′-GGGGACAAGTTTGTACAAAAAAGCAGGCTACCACCATGCA GCTGAGGAA CCCAGA-3′, *GLA* antisense: 5′-GGGGACCACTTTGTACAAGAAAGCTGGGTC TTAAAGTAAGTCTTTTAATGACATC-3′, *NAGA* sense:5′-GGGGACAAGT TTGTACA AAAA AGCAGGCTACCACCATGCTGCTGAAGACAGTGCTC-3′, *NAGA* antisense:5′-GGGGACCA CTTTGTACAAGAAAGCTGGGTCTCAGCTGGGACATCTCCAG-3′. The elements were cloned via Gateway cloning into the donor vector pDONR221 (BP reaction), following transformation of DH5α *E. coli* strain. Clones were collected upon ampicillin selection and their sequences were confirmed. Next, they were cloned into the destination vector, pcdna-DEST40-zeo (LP reaction), forming the final construct to transfect HEK293 cells. The HEK293 cells were cultured in six well plates in 2.5 ml Dulbecco’s Modified Eagles Medium (DMEM, Sigma) supplemented with 10% fetal calf serum, 1% penicillin/streptomycin and glutamax at 37°C, at 7% CO_2_. Three μg of pcdna-DEST40-zeo DNA was transfected into cultured HEK293 cells using polyethylenimine, primarily mixed with serum free DMEM medium. After 2 days of cell culture, 200 μg/μl of zeocin was added to the culture media for selection of clones harboring the gene of interest. The cells grew for 3 weeks at 37°C, at 7% CO_2_ in the presence of antibiotics and lysed in 20 mM KPi buffer (pH 6.5), 0.1% Triton with additional protease inhibitor by Roche. The cells were stored at -150°C in DMEM, 20% fetal calf serum, 10% DMSO until further use.

### Enzymatic Activity Measurements

Both α-galactosidase A and α-*N*-acetyl-galactosaminidase activities of samples were examined with corresponding 4-methylumbelliferyl (4MU) substrates. For α-galactosidase activity measurement samples were incubated for 1 h at 37°C with a final concentration of 1.2 mg/ml 4MU-α-D-galactosylpyranoside (4MU-α-GAL) in 150 mM citrate-phosphate buffer pH 4.6, supplemented with 0.1% (w/v) BSA and released 4MU was quantified as described earlier (Blom et al., 2003).

Activity of plant produced recombinant enzymes and Fabrazyme toward 5 μM of NBD-C12-Gb3 substrate was measured for 3 h at 37°C in 150 mM citrate-phosphate buffer (pH 4.6) containing 0.05% (v/v) Triton X-100 and 0.2% (w/v) sodium taurocholate, pH 4.6. Lipids were extracted with the Bligh and Dyer procedure, applied to HPTLC. The plate was scanned for fluorescent lipids with a Typhoon FLA 9500. Protein concentrations in assay were for Fabrazyme 3.8 μg/ml, α-GAL 3.2 μg/ml, α-NAGAL 40 μg/ml, and α-NAGAL^EL^ 16 μg/ml. Activity of recombinant enzymes toward natural C18: Gb3 was measured using 500 pmol of lipid in (100 μl total volume) 150 mM citrate-phosphate buffer with 0.05% (v/v) Triton X-100 and 0.2% (w/v) sodium taurocholate for 3 h at 37°C. The pH was 4.2 for α-GALs and 5.2 for α-NAGALs. After incubation, neutral lipids were extracted by the Folch method. Deacetylation of lipids was performed as described earlier (Ferraz et al., 2016b). After deacetylation, samples were dried and further cleaned by adding 1:1 butanol: water. The butanol phase was collected, dried and lipids were dissolved in 200 μl methanol, ready for LC–MS/MS injection. The conversion of Gb3 to lactosylceramide (Lac Cer) was measured in LC–MS/MS using C17-Gb3 and C17-Lac Cer as controls and C17-Sphinganine as internal standard. The same assay conditions were used when applying natural Lyso-Gb3 as substrate. Lipids were extracted according to Bligh and Dyer and subjected to LC–MS/MS injection as above. Isotope ^13^C-Lyso-Gb3 was used as an internal standard for monitoring the conversion of Lyso-Gb3 into Lyso-Lac Cer. Protein concentrations in assays were for Fabrazyme 1.9 μg/ml, α-GAL 1.6 μg/ml, α-NAGAL 20 μg/ml, and α-NAGAL^EL^ 8 μg/ml.

### Determination of Kinetic Parameters

*K_m_, V_max,_* and *k_cat_* values were determined using 4MU substrates. Reactions were performed for 1 h or 30 min at 37°C at 10 different 4MU-α-GAL and 4MU-α-NAGAL concentrations in 150 mM citrate-phosphate buffer pH 4.6 supplemented with 0.1% (w/v) BSA. The 4MU-α-GAL concentrations in the assays ranged from 0.074 to 4.72 mM; for 4MU-α-NAGAL from 0.022 to 0.91 mM. Protein concentrations in the assays were: for α-GAL 7 ng/ml, Fabrazyme 2 ng/ml, α-NAGAL 16 ng/ml, when using 4MU-α-NAGAL substrate and 100 ng/ml, when using 4MU-α-GAL substrate, and for α-NAGAL^EL^ 16 ng/ml. Parameters were calculated using GraphPad Prism6.

### *N*-glycan Analysis of Plant Produced α-Galactosidases

Proteins were deglycosylated with EndoH and PNGase F (Wilbers et al., 2016). MALDI-TOF analysis performed as previously described (Wilbers et al., 2016).

### Sodium Dodecyl Sulfate-Polyacrylamide Gel Electrophoresis

To examine purity and molecular mass of recombinant α-galactosidases, sodium dodecyl sulfate-polyacrylamide gel electrophoresis (SDS- PAGE) (10% polyacrylamide) was used (Witte et al., 2010). Samples were run under reduced conditions. After SDS-PAGE analysis, gels were stained with Coomassie Brilliant Blue staining or silver stain using the PhastSystem system (GE Healthcare).

### ABP-Based Fluorescence Staining

The α-GAL Cy5 ABP was diluted in 150 mM citrate-phosphate buffer, pH 5 and applied to lysates or pure protein preparations at 0.5 or 0.25 μM final concentration, respectively. The samples were incubated for 30 min, at 37°C and the reaction was stopped via the addition of 4x gel loading buffer (containing β-mercaptoethanol). The samples were then boiled for 5 min at 95°C and loaded on 10% SDS-PAGE gels. Subsequently, western blotting and fluorescence scanning was performed exactly as described earlier (Witte et al., 2010; Willems et al., 2014).

### Protein Determination

Protein concentrations were determined with a Micro BCA Protein Assay Reagent Kit (PIERCE), and bovine serum albumin was used as a standard, according to the supplier’s protocol. In addition, pure protein concentrations were measured in Nano Drop 2000c (Thermo Scientific) via adjusting the molecular weight and extinction coefficient parameters.

### Detection of Cross Reactivity with Antibody (Ab)-Positive FD Serum

Samples were incubated for 1 h 4°C while rolling, with 2 μl Ab^+^ FD serum or normal serum of an FD patient and α-galactosidase activity was measured with 4MU-α-GAL and 4MU-α-NAGAL substrate (Linthorst et al., 2004).

### Degradation of Lyso-Gb3 in FD Serum

To serum from two different FD patients pre-ERT, recombinant enzymes (8 μmol artificial substrate hydrolysis/h/ml) were added and incubated overnight at 37°C. Then, lipids were extracted as described before and applied to LC–MS/MS.

## Results

### Transient Production of Human α-Galactosidases in *N. benthamiana* Leaves

**FIGURE 1:**
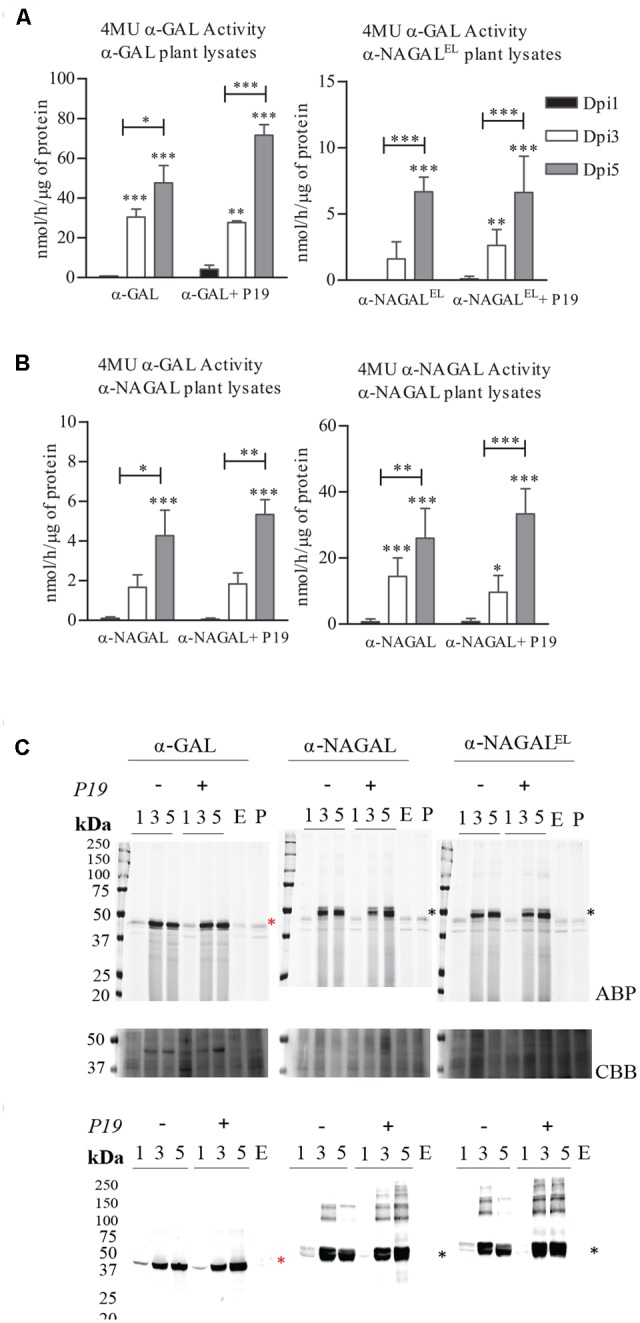
Production of α-galactosidases α-GAL, α-NAGAL and α-NAGAL^EL^ in transiently transformed *Nicotiana benthamiana* leaves. Samples were harvested 1, 3, and 5 days post-infiltration (dpi) either or not co-expressed with P19 silencing inhibitor. **(A)** Enzymatic activity was measured with corresponding 4MU-substrate (Represented in black the 4MU activity of lysates harvested in 1st Dpi, light gray 3rd Dpi, dark gray 5th Dpi) (*n* = 4, error bars indicate mean ± standard deviation). Asterisk(s) indicate significant differences as measured by a two-way ANOVA corrected using Bonferroni *post hoc* tests, ^∗^*P* < 0.05, ^∗∗^*P* < 0.01, ^∗∗∗^*P* < 0.001). **(B)** ABP detection of active enzyme molecules present in plant lysates. As controls an empty vector sample (E.V) and an untreated plant leaf (P.L). The gels were stained with Coomassie Brilliant Blue staining (CBB) as a loading control. **(C)** Western blot analysis of the plant produced α-galactosidases using anti-α-GAL and anti-NAGA polyclonal rabbit antibodies raised against the human homolog. As control an empty vector sample (E). Black asterisk indicating the α-NAGALs and red asterisk the α-GAL.

We first established the optimal conditions for production of α-galactosidases α-GAL, α-NAGAL, and α-NAGAL^EL^ in a transient expression system in *N. benthamiana*. In the constructs the sequences of mature α-galactosidases were preceded by that of the *Arabidopsis thaliana* chitinase signal peptide. *Agrobacterium tumefaciens* infiltrated leaves of 5–6 weeks old *N. benthamiana* plants were collected at 1, 3, and 5 days post-infiltration (dpi), lysates were prepared and enzymatic activities of α-galactosidases were measured using the respective 4MU substrate (**Figure 1A**). For each enzyme, an optimal yield was observed at 5 dpi, while co-expressing the p19 silencing suppressor of tomato bushy stunt virus. This time point was used throughout all later experiments for harvesting. The amounts of active recombinant α-GAL, α-NAGAL and α-NAGAL^EL^ at 5 dpi was 71, 5 and 7 nmol/h/μg total protein, respectively, as measured with 4MU-α-GAL substrate. No 4MU-α-NAGAL activity was detected for the α-NAGAL^EL^ enzyme, whereas the wild type reached 33 nmol/h/μg at 5 dpi (**Figure 1A**). The amount of enzyme activity was proportional to the amount of active enzyme molecules detected with the fluorescent ABP (**Figure 1B**) and that detected with western blotting using anti-α-GAL and α-NAGAL antibodies (**Figure 1C**). The molecular weight of α-GAL was about 44 kDa. Those of the two α-NAGALs were about 49 kDa, the higher mass being due to the known presence of one extra *N*-glycan (Clark and Garman, 2009).

### Purification and Biochemical Characterization of Plant Produced α-Galactosidases

**FIGURE 2:**
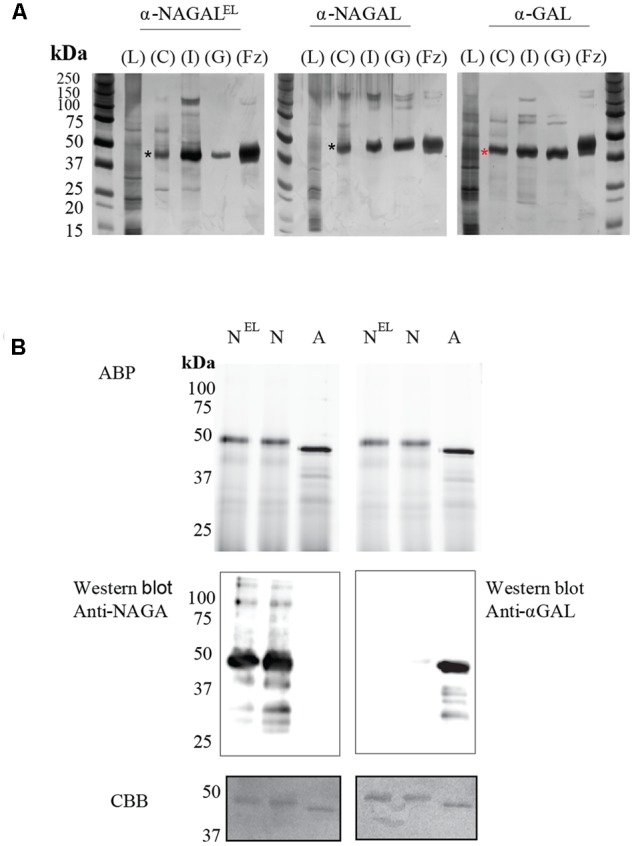
Overview of enzyme purifications: activity based probe detection, Immunoblotting and Coomassie Brilliant Blue, staining of the plant produced pure enzymes. **(A)** SDS-PAGE and silver staining of fractions obtained during purification. Four μg of total protein per lane was loaded for all unpurified samples and 1–2 μg for all pure protein fractions (α-NAGAL, α-NAGAL^EL^, and α-GAL). Shown are: starting material/Lysate (L), bound protein to Concanavalin A (C), pooled collected eluate of ion exchange (I), and the final pooled gel filtration fraction (GL) with highest enzyme specific activity. For comparison is shown recombinant α-galactosidase A, Fabrazyme (Fz). Black asterisk indicating the α-NAGALs and red asterisk the α-GAL. **(B)** Before electrophoresis, 1 μg of each pure enzyme was treated with 0.25 μM of Cy5 α-galactosidase activity based probe, ABP. N^EL^ = α-NAGAL^EL^, N = α-NAGAL, A = α-GAL. The gels were scanned λaexc = 635 nm, then immunoblotted with anti-α-GAL or anti-NAGAL rabbit polyclonal antibodies, following Coomassie Brilliant blue, CBB, staining of the blots. The same gel had to be repeated since anti-α-GAL and anti-NAGA antibodies were both polyclonal anti-rabbit.

Recombinant α-galactosidases were purified from lysates of *N. benthamiana* leaves by sequential chromatography during which the presence of enzymes was monitored by measurement of enzymatic activities with 4MU-substrates. As generic first step we used Concanavalin A-Sepharose chromatography exploiting the presence of mannose-containing *N-*glycans in the recombinant α-galactosidases ensuring high affinity binding to the immobilized lectin (Yasuda et al., 2004). The recovery in this step was high (>40%) for each enzyme and led to >50-fold purification (**Table 1**). The next step in purification was ion exchange chromatography performed at pH 5.0 for α-GAL and at pH 6.3 for α-NAGAL and α-NAGAL^EL^ as described in experimental procedures. This led for each enzyme to considerable further purification with acceptable recovery (**Table 1**). The final purification step for all enzymes was gel filtration, resulting in an apparently pure protein as judged by silver staining of protein resolved by SDS-PAGE (**Figure 2A**). About 1 μg α-GAL, α-NAGAL, and α-NAGAL^EL^ was purified from 1 mg of soluble lysate protein.

**Table 1:** Overview of enzyme purifications.

Enzymes	Purification steps	Volume (ml)	Total protein (mg)	Total activity (μmol/h)	Total specific activity (μmol/h/mg)	Purification fold	% Yield = recovery of activity
α-GAL	Plant extract	49	136	3412	25	0	100
	Con-A	3	1.3	1928	1444	57	57
	Cation exchange	2	0.4	860	2151	86	25
	Gel filtration	1.4	0.12	308	2585	103	9

α-NAGAL	Plant extract	38	135	319	2.3	0	100
	Con-A	3	1	112	107	45	35
	Anion exchange	4	0.7	94	132	56	30
	Gel filtration	2	0.14	29.5	211	89	9

α-NAGAL^EL^	Plant extract	25	64	314	5	0	100
	Con-A	2	0.45	122	270	55	49
	Anion exchange	4	0.2	55	273	56	17
	Gel filtration	1.2	0.056	28	501	102	9

The apparent molecular weights of all three α-galactosidases as determined by gel filtration coincided with dimers (113 kDa for α-GAL, 134 kDa for α-NAGAL, and 132 kDa for α-NAGAL^EL^) (Supplementary Figures S2A,B). Of note, the specific activity of plant-produced α-GAL (2.35 mmol/h/mg) was quite comparable to that of Fabrazyme (2.18 mmol/h/mg) (**Table 2**) (Tajima et al., 2009). After purification, further enzyme characterization was performed. Equal μg amounts of pure enzymes were incubated with fluorescent ABP followed by SDS-PAGE and fluorescence scanning analysis (**Figure 2B**). The intensity of the fluorescently labeled bands was the same for the three enzymes, revealing similar amounts of active enzyme molecules in each purified protein preparation. Western blotting using anti-α-GAL and anti-α-NAGAL antibodies showed that α-NAGAL^EL^ was not recognized by anti-α-GAL antibody (**Figure 2B**).

**Table 2:** Specific activities as determined by 4MU substrates.

	4MU-α-GAL specific activity (mmol/h/mg)	4MU-α-NAGAL specific activity (mmol/h/mg)
α-GAL	2.352	<0.05
α-NAGAL	0.192	1.33
α-NAGAL^EL^	0.547	<0.05
Fabrazyme	2.184	<0.05

### *N*-glycan Profile of the Plant Produced Enzymes

**FIGURE 3:**
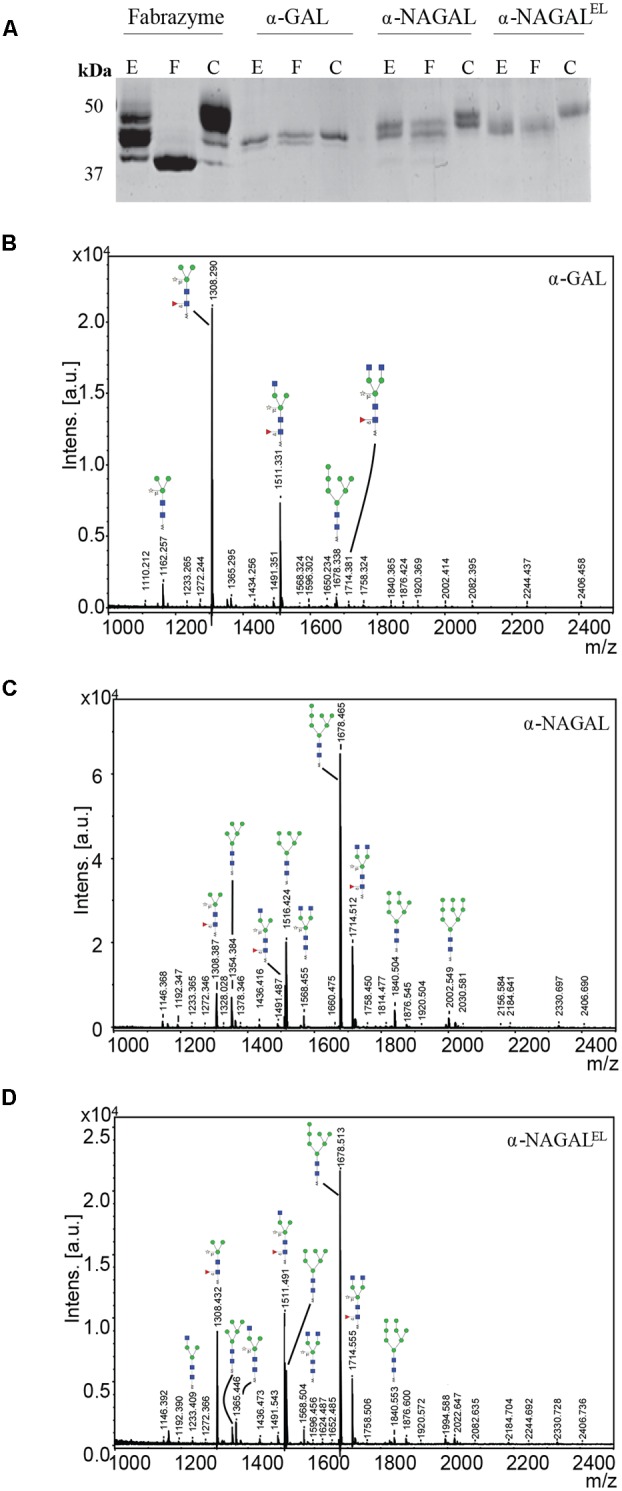
*N*-glycans of α-galactosidases. **(A)** SDS-PAGE analysis followed by Coomassie Brilliant Blue staining of 1 μg pure plant produced proteins and 3 μg of Fabrazyme. E = protein treated with Endo H, F = protein treated with PNGase F, C = untreated protein. **(B–D)** *N*-glycosylation profiles were determined by MALDI-TOF MS analysis of 2-aminobenzoic acid derivatised PNGase-A released *N*-glycans of plant produced galactosidases. Glycan structures depicted were deduced from the measured m/z values. Blue square, *N*-acetylglucosamine; red triangle, fucose; open star, xylose; green circle, mannose. Presented *N*-glycan profiles of the following proteins: **(B)** α-GAL, **(C)** α-NAGAL, **(D)** α-NAGAL^EL^.

Next, the *N*-glycan composition of the purified α-galactosidases was examined using deglycosylation with Endo H and PNGase F endoglycosidases following SDS-PAGE analysis. Fabrazyme, produced in CHO cells, was used as a control. Consistent with literature reports, PNGase F led to conversion of Fabrazyme to a molecular mass of 39 kDa (**Figure 3A**). In the case of plant produced enzymes the reductions in molecular mass with EndoH or PNGase F were less pronounced (**Figure 3A**). This difference can be attributed to the presence of α1-3 core fucose residues, attached to most of the *N*-acetyl-glucosamine backbones (GlcNAc), inhibiting digestion by the endoglycosidases. The higher molecular weight of Fabrazyme compared to the plant produced enzymes could be due to the different *N*-glycan composition of this enzyme, mainly consisting of complex *N*-glycan structures, with additional mannose 6 phosphate residues (Sakuraba et al., 2005). To get more insight in the *N*-glycan composition of the plant produced enzymes, all *N*-linked glycans were released with PNGase A (not inhibited by the presence of α1-3 fucose) and subjected to MALDI-TOF MS (**Figures 3B–D**). The most prominent *N-*glycan type of α-GAL recombinant protein was paucimannose structure (Man_3_GlcNAc_2_) with α1-3 fucose attached on the core GlcNAc and β1-2 xylose on the first mannose. Conceivably, the enzyme was secreted to the apoplastic fluid and hexosaminidases cleave the GlcNAc molecules yielding the pausimanosidic structure. On the other hand, both α-NAGALs have oligo-mannose type *N*-glycans (Man_7_GlcNAc_2_) as the most abundant *N*-glycan type, suggesting their retention in Golgi and /or ER compartments.

### Cross Reactivity with Antibodies (Ab) in Fabry Serum and Stability Tests

**FIGURE 4:**
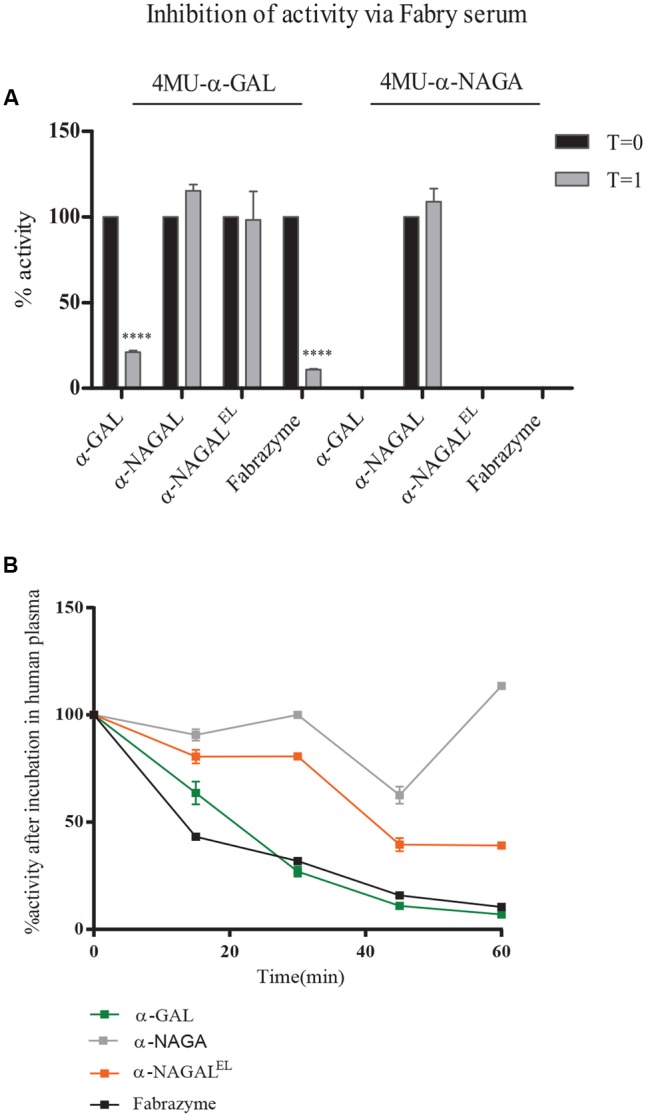
Inhibition of activity of α-galactosidases by Ab-positive Fabry serum. **(A)** Percentage inhibition by FD serum, obtained after 6 years ERT (T = 1), of enzymatic activity measured with 4MU-α-GAL and 4MU-α-NAGAL. As negative control, serum of FD patient pre-ERT (T = 0), was used. Asterisk(s) indicate significant difference as measured by a two-way ANOVA, ^∗∗∗∗^*P* < 0.0001. **(B)** Percentage activity after incubation of 1 μg/ml pure enzyme in human plasma for 15, 30, 45, and 60 min.

We next examined the cross reactivity of recombinant α-galactosidases toward neutralizing antibodies in the serum of an Ab-positive male FD patient receiving ERT for 6 years (T = 1). As a control, serum was used of the same patient before ERT (T = 0) and still lacking antibodies against therapeutic α-GAL. Inhibition of enzymatic activity by presence of Ab-positive serum was determined (**Figure 4A**). Activity of plant produced α-GAL and Fabrazyme was clearly inhibited, but activities of α-NAGALs, as determined with 4MU-α-GAL and/or 4MU-α-NAGAL substrates were not significantly influenced. The stability of the plant-produced galactosidases and Fabrazyme in human plasma was next studied by incubating enzymes at 37°C for different time periods and detection of residual enzymatic 4MU-α-GAL activity (**Figure 4B**). Alpha-NAGAL^EL^, like α-NAGAL, was more stable than both α-GALs (**Figure 4B**). The finding was made several times with independent incubations.

### Enzymatic Activities of Plant Produced α-GAL, α-NAGAL, and α-NAGAL^EL^ toward Artificial Substrate(s) and Their Comparison with the Same Enzymes Produced in HEK293 Cells

**FIGURE 5:**
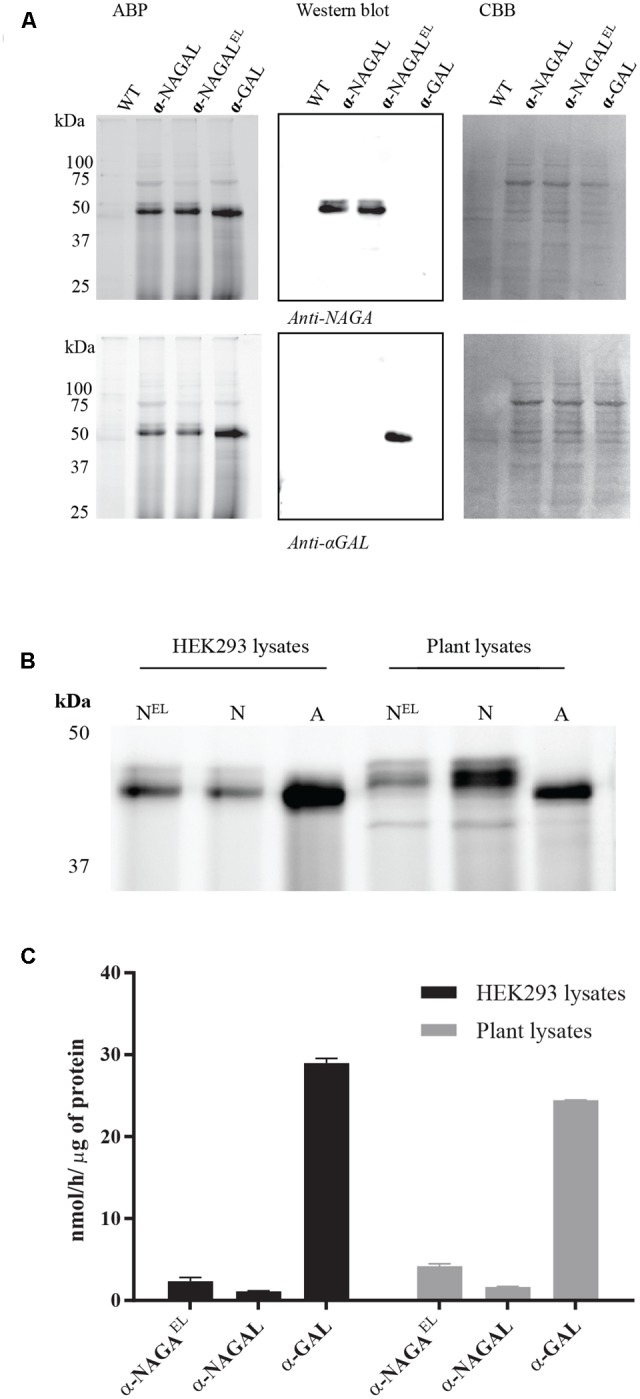
Comparison of the biochemical properties of recombinant galactosidases produced in plants and HEK293 cells. **(A)** Overexpression of human galactosidases in HEK293 cells was estimated via SDS-PAGE analysis. 20 μg of total soluble protein were incubated with 0.5 μM of Cy5 ABP, following Western blot analysis and Coomassie Brilliant Blue, CBB, staining of the blot. **(B)** ABP labeling of active enzyme molecules present in HEK293 and plant lysates overexpressing the proteins α-NAGAL^EL^, α-NAGAL, and α-GAL. Different total protein content was loaded in each lane. **(C)** The 4MU-α-GAL specific activities of HEK293 and plant lysates overexpressing the human galactosidases.

We next studied the substrate specificity of the various recombinant α-galactosidases. First, activity was measured toward artificial 4MU-α-GAL and 4MU-α-NAGAL substrates. The amino acid substitutions Ser188Glu and Ala191Leu in the catalytic pocket of α-NAGAL (α-NAGAL^EL^) abolished activity toward 4MU-α-NAGAL as earlier reported (Tajima et al., 2009; Tomasic et al., 2010) (**Table 2**). The noted increase in hydrolytic specific activity toward 4MU-α-GAL (about a doubling) was less prominent as earlier reported by Tajima et al. (2009). The kinetic parameters kcat and Km determined with 4MU-α-GAL as substrate were improved by the amino acid substitutions in α-NAGAL^EL^ (**Table 3**). The plant produced α-GAL showed comparable kinetic parameters to Fabrazyme (**Table 3**) (Hopkin et al., 2003; Lee et al., 2003). To determine whether production in *N. benthamiana* leaves influences kinetic features, the various recombinant enzymes were also generated in human HEK293 cells as described in experimental procedures (**Figure 5A**). ABP-labeling showed that similar proteins were produced in *N. benthamiana* and HEK293 cells (**Figure 5B**). The specific activity toward 4MU-α-GAL was also comparable, being relatively low for α-NAGAL and increased (threefold) by the amino substitutions in α-NAGAL^EL^ (**Figure 5C**).

**Table 3:** Michaelis-Menten kinetics as determined by 4MU substrates.

	4MU-α-GAL				4MU-α- NAGAL			
	Km (mM)	Vmax (mmol/h/mg)	kcat (s^-1^)	kcat/Km (mM/s^-1^)	Km (mM)	Vmax (mmol/h/mg)	kcat (s^-1^)	kcat/Km (mM/s^-1^)
α-GAL	2.478	4.219	55.96	22.58	No activity detected			
α-NAGAL	6.02	0.527	6.89	1.14	0.7	3.9	25.51	36.13
α-NAGAL^EL^	3.18	0.71	14.52	4.79	No activity detected			
Fabrazyme	2.431	3.954	52.11	21.43	No activity detected			

### Activity of Recombinant Enzymes toward (Semi) Natural Substrates

**FIGURE 6:**
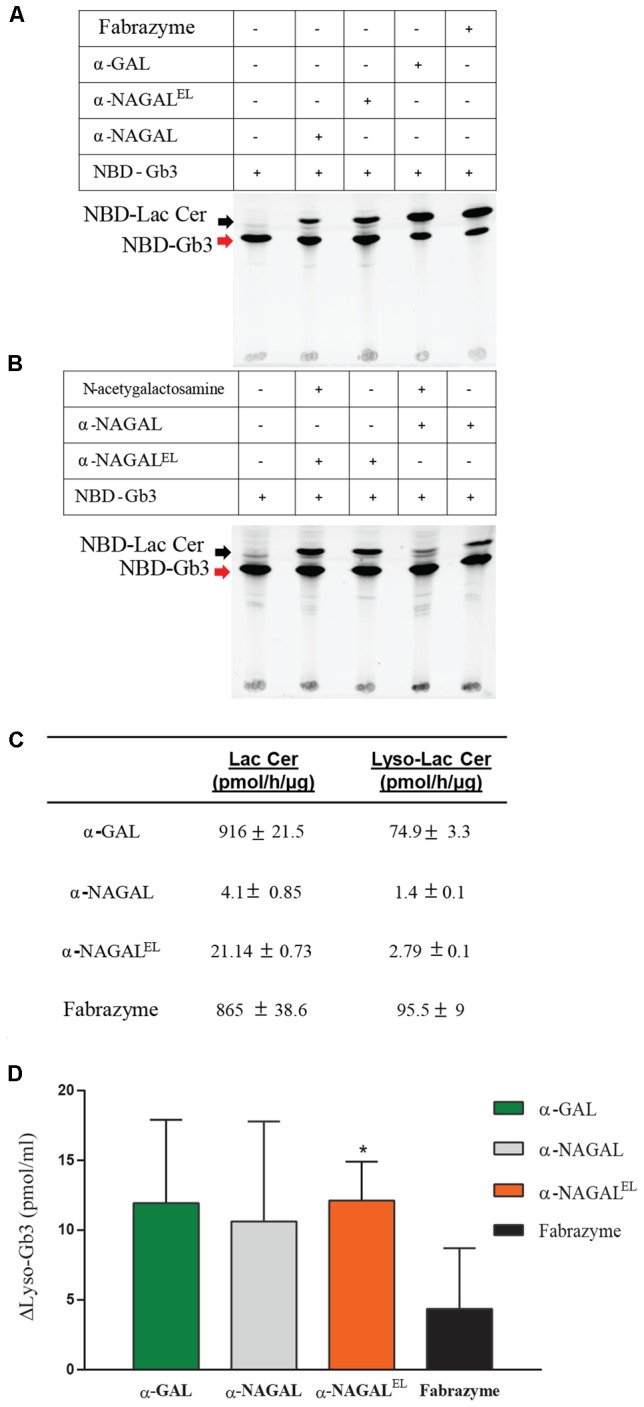
Activity of α-galactosidases toward semi-natural and natural lipid substrates. **(A)** Pure recombinant proteins were incubated with 5 μM of NBD-C12-Gb3. detection of formation of NBD-C12Lactosylceramide, Lac Cer detected by HPTLC. **(B)** Pure α-NAGALs (wild type and modified) were incubated with 5 μM of NBD-C12-Gb3 in presence/absence of 2.5 mM *N*-acetyl-galactosamine. Formation of NBD-C12-Lactosylceramide detected by HPLC. **(C)** Pure plant produced galactosidases and Fabrazyme were incubated with 5 μM of C18-Gb3, and Lyso-Gb3. Formation of Lac Cer and Lyso-Lac Cer (pmol/h/μg) detected by LC–MS/MS. Data represent the mean ± SD, *n* = 2. **(D)** Correction of Lyso-Gb3 in FD serum of two different Fabry patients pre-ERT after incubation with pure plant produced enzymes (each 8 μmol artificial 4MU-substrate hydrolysis/h/ml) overnight at 37°C. Lyso-Gb3 levels measured by LC–MS/MS. Data represent as mean ± SD, *n* = 2. Asterisk(s) indicate significant difference as measured by a standard Student’s *t*-test, ^∗^*P* < 0.05.

The activity of plant produced galactosidases toward lipid substrates was next determined. NBD-Gb3 was found to be degraded by Fabrazyme and plant-produced α-GAL on a par (**Figure 6A**). Of note, significant degradation of NBD-Gb3 to NBD-Lac Cer by α-NAGAL and α-NAGAL^EL^ was detected (**Figure 6A**). To further substantiate the findings, we incubated wild type and mutant α-NAGALs with NBD-Gb3 in the absence or presence of 2.5 mM *N*-acetylgalactosamine, a potent inhibitor of α-NAGAL activity (**Figure 6B**). The wild type α-NAGAL was inhibited, resulting in loss of NBD-Lac Cer formation, whereas α-NAGAL^EL^ was not. Next, activity toward 5 μM of natural Gb3 and its deacetylated form Lyso-Gb3, was determined using detection of metabolites with LC–MS/MS. The analysis revealed a 2–5-fold increased activity of α-NAGAL^EL^ compared to α-NAGAL with both substrates (**Figure 6C**). The pH optimum was pH 4.2 for α-GALs and pH 5.2 for both α-NAGALs (Supplementary Figure S3).

Finally, serum of male FD patients, containing high amount of Lyso-Gb3, was incubated overnight with recombinant galactosidases (at a concentration of 8 μmol/h/ml activity mimicking levels reached during ERT). Of note, the rate of degradation of Lyso-Gb3 in the serum sample was highest with α-NAGAL^EL^ (**Figure 6D**). Likely this reflects the greater stability and ongoing activity of α-NAGAL^EL^ at neutral pH as compared to that of α-GALs.

## Discussion

The combined challenges of high costs, immunogenicity and poor efficacy posed by present Fabry ERT’s prompted us to consider an examination of wild type and mutant recombinant human α-galactosidases. *N. benthamiana* leaves, as production system, allowed expression of all enzymes at substantial amounts, 0.1–0.2% of total soluble protein. We used agro-infiltration of *N. benthamiana* leaves for the production of the enzymes as a reliable, low cost and fast method for transient gene expression. *N. benthamiana* is a rapidly growing plant species, which leaves can be easily infiltrated with *Agrobacterium tumefaciens* harboring the genes encoding the enzymes to be produced. In addition, a high variety of expression vectors available for *Nicotiana* plants provide another advantage of using this species for recombinant protein production (Chen et al., 2013; Leuzinger et al., 2013). Further, the composition of *N*-glycans can be tailored in *N. benthamiana* variants (Wilbers et al., 2016). Stable expression of the glycosidases might be considered to eliminate inter-batch yield variations. *A priori* the production in another “plant” host might also be considered, for example stable expression in lettuce (*Lactuca sativa*), algae or moss, in order to conceivably enhance yields and to avoid the high levels of phenolic and toxic alkaloids present in *Nicotiana* plants that might affect the purification procedures (Hempel et al., 2011; Chen et al., 2013; Xu and Zhang, 2014; Reski et al., 2015). Furthermore, recombinant human α-GAL has earlier been successfully produced in cultured *Nicotiana tabacum* cells and in an engineered moss cell line, eliminating possible transgene flow that might occur while using whole plants as production platforms (Kizhner et al., 2014; Shen et al., 2016).

The presently expressed α-GAL seems most likely to be secreted or transported to the vacuole as based on its *N*-glycan composition consisting largely of Man3GlcNAc2 with α1-3 fucose attached to the core GlcNAc and β1-2 xylose to the first mannose (Castilho and Steinkellner, 2012). In contrast, the transiently expressed α-NAGALs show prominent Man7GlcNAc2 *N*-glycans, more consistent with retention in the *cis* Golgi or ER (Castilho and Steinkellner, 2012). Some bias might be introduced by the lectin purification step. However, the recovery of enzymes in the lectin binding step is high, minimizing the bias. The observed specific activities of the purified enzymes were in the range of single digit nanomole substrate hydrolysis/μg pure protein per hour, being comparable to that of Fabrazyme. Recombinant, plant produced galactosidases were all labeled by fluorescent α-galactosyl cyclophellitol-aziridine ABP. The intensity of ABP labeling per protein amount was identical for all galactosidases, substantiating further that in all cases the majority of purified protein is enzymatically active.

The observed specific activities of the produced α-galactosidases warrant discussion. The specific activity of pure α-NAGAL^EL^ (0.55 mmol/h/mg) as measured with 4MU-α-GAL was 2–3-fold increased to that of wild type α-NAGAL (0.19 mmol/h/mg). The Km of α-NAGAL^EL^ (3 mM) was lower compared to that of α-NAGAL (6 mM). Almost the same specific activity for α-NAGAL^EL^ (0.5 mmol hydrolysis/h/mg) was reported by Sakuraba and co-workers (Tajima et al., 2009). They related this to that of wild type α-NAGAL from a different source, resulting in an apparent 43-fold improved α-galactosidase activity imposed by the introduction of the S188E and A191L in α-NAGAL (Tajima et al., 2009). Our results are in closer alignment with those of Garman and co-workers (Tomasic et al., 2010). Using p-nitrophenyl-α-Gal as substrate the kcat/Km value of α-NAGAL^EL^ was found to be 4.6-fold higher than that of α-NAGAL, similar to the fourfold increase of kcat/Km value with 4MU-α-GAL substrate observed by us (Tomasic et al., 2010). Comparison of plant produced α-GAL with Fabrazyme revealed that their specific activities toward 4MU-α-GAL, as well as Gb3, are almost identical. We also produced the various recombinant α-galactosidases in mammalian HEK293 cells showing similar kinetic parameters as the plant-produced enzymes, indicating that these are not influenced by the production platforms.

Fabrazyme produced in CHO cell line contains the sialic acid *N*-glycolylneuraminic which does not occur in humans and therefore is potentially immunogenic (Bekri, 2006; Castilho and Steinkellner, 2012; Kizhner et al., 2014, 2016). The plant-produced recombinant enzymes of the current study do not contain *N*-glycolylneuraminic acid in their *N*-glycans. The *N*-glycan profiles of all plant-produced recombinant enzymes were relatively homogenous, but differences were seen in *N*-glycans among the various enzymes. Most importantly, the majority of *N*-glycans of all recombinant enzymes were found to be core α1-3 fucosylated and β1-2 xylosylated. The same modifications occur in *N*-glycans of carrot-produced taliglucerase (human glucocerebrosidase) (Shaaltiel et al., 2007). Of note, there is no evidence that these modifications induce immune responses in GD patients (Shaaltiel et al., 2007). *A priori* this does not exclude immunogenicity in FD patients.

The recombinant enzymes produced in *N. benthamiana* lack M6P residues. In theory, the glycans may be swapped for other structures through the chemoenzymatic methodology developed by Fairbanks and colleagues (Priyanka et al., 2016). However, given the limited success of existing Fabry ERT aiming at M6P receptor mediated delivery of therapeutic enzyme to lysosomes it might be considered to refrain from this type of enzyme targeting. We earlier demonstrated that deacylated Gb3, Lyso-Gb3, is extremely elevated in FD patients and α-galactosidase A deficient mice (Aerts et al., 2008; Ferraz et al., 2016b). Excessive Lyso-Gb3 has been shown to be toxic for nociceptive peripheral neurons and podocytes in FD patients (Choi et al., 2015; Sanchez-Niño et al., 2015). Reduction of Lyso-Gb3 in FD patients therefore seems a valid therapeutic goal. In view of this consideration above, our finding that not only α-GAL but also α-NAGALs are able to degrade Gb3 and Lyso-Gb3 is of interest. The amino acid substitutions in α-NAGAL^EL^ improve ∼2-fold the capacity of the enzyme to degrade Lyso-Gb3 and ∼5-fold that for Gb3. The relative efficacy of α-NAGAL^EL^ compared to α-GAL in ability to degrade Lyso-Gb3 in FD serum is even better. The greatest reduction of Lyso-Gb3 is detected with α-NAGAL^EL^ added to FD serum at 37°C. The superior action of α-NAGAL^EL^ is likely due to its stability at neutral pH. This remarkable finding suggests that the use of non-antigenic (modified) α-NAGAL might be a way to reach desired reductions in circulating Lyso-Gb3. The use of (modified) α-NAGALs benefits from the higher stability and activity of the enzyme at neutral pH as compared to α-GAL. PEGylation could be employed to increase the presence of (modified) α-NAGAL in the blood. Of note, endogenous α-NAGAL in the circulation of normal individuals and FD patients is relatively low (Aerts, unpublished observations) and therefore likely does not contribute significantly to metabolism of the glycosphingoid base in the blood.

## Conclusion

We here demonstrate that production of substantial amounts of wild type human α-GAL, α-NAGAL and modified α-NAGAL^EL^ is feasible in *N. benthamiana*, resulting in enzymes with comparable kinetic properties to those produced in mammalian cell production systems. The introduction of two amino acid substitutions in α-NAGAL improves its α-galactosidase activity as measured with artificial fluorogenic substrate and natural lipids. A significant difference between α-GAL and α-NAGAL is the greater stability and activity at neutral pH of the latter enzyme. Furthermore α-NAGAL is not inhibited by existing Ab in male FD patients by present ERTs. Modifications in the catalytic pocket of α-NAGAL can improve its capacity to degrade Gb3 and Lyso-Gb3. The kinetic properties of modified α-NAGAL and its greater stability at neutral pH could be further exploited for treatment of FD. In particular, use of modified α-NAGAL to reduce circulating Lyso-Gb3 in FD patients warrants further examination.

## Author Contributions

KK designed and conducted the majority of the experiments and wrote the manuscript together with JA. TB produced the Cy5 ABP, also contributed in writing of the manuscript, supervised by HO. LW helped in the design of plant expression vectors and plant infiltrations. CH conducted the *N-*glycan analysis. GM and NG supervised the purification procedures. MdG and WK characterized the ABP made by TB. RB made the HEK293 constructs. MM and MF contributed in the lipid analysis. AS and DB were involved in the design of the research idea and contributed in writing of the manuscript. JA supervised the entire work, conceived the experiments together with KK and wrote the manuscript.

## Conflict of Interest Statement

The authors declare that the research was conducted in the absence of any commercial or financial relationships that could be construed as a potential conflict of interest.
