# Supplementary material for: Human Alpha Galactosidases Transiently Produced in Nicotiana benthamiana Leaves: New Insights in Substrate Specificities with Relevance for Fabry Disease
Source: Front Plant Sci. 2017 Jun 21;8:1026. doi: 10.3389/fpls.2017.01026 (PMC5478728; doi:10.3389/fpls.2017.01026)
Supplement: Supplementary file 1 [file Presentation_1.PDF]

Supplementary Material

Human alpha galactosidases transiently produced in *Nicotiana benthamiana* leaves: new insights in substrate specificities with relevance for Fabry disease

Kassiani Kytidou[1], Thomas J.M. Beenakker[2], Lotte B. Westerhof[3], Cornelis H. Hokke[4], Geri F. Moolenaar[5], Nora Goosen[5], Mina Mirzaian[1], Maria J. Ferraz[1], Mark de Geus[1] ,Wouter W. Kallemeijn[1], Herman, S. Overkleeft[2], Rolf G.Boot[1], Arjen Schots[3], Dirk Bosch[3], Johannes M.F.G. Aerts[1*].

1. Department of Medical Biochemistry, Leiden Institute of Chemistry, Einsteinweg 55, 2333 CC Leiden, The Netherlands

2. Department of Bio-organic Synthesis, Leiden Institute of Chemistry, Einsteinweg 55, 2333 CC Leiden, The Netherlands

3. Wageningen University and Research, Plant Sciences Group, Droevendaalsesteeg 1, 6708 PB Wageningen, The Netherlands

4. Department of Parasitology, Centre of Infectious Diseases, Leiden University Medical Center, Albinusdreef 2, 2333 ZA Leiden, The Netherlands

5. Cloning and Protein Purification Facility of Leiden Institute of Chemistry, Einsteinweg 55, 2333 CC Leiden, The Netherlands

Netherlands

* Corresponding author: j.m.f.g.aerts@lic.leidenuniv.nl

Synthesis and characterization of activity-based probe α-galactopyranose-configured cyclophellitol aziridine tagged with fluorescent Cy-5.

Synthesis

All chemicals were purchased from Acros, Sigma Aldrich, Biosolve, VWR, Fluka, Merck and Fisher Scientific and used as received unless stated otherwise. Dichloromethane (DCM), *N*,*N*-dimethylformamide (DMF) and toluene were stored over flame-dried 4 Å molecular sieves before use. Traces of water from reagents were removed by co-evaporation with toluene in reactions that require anhydrous conditions. All reactions were performed under an argon atmosphere unless stated otherwise. TLC analysis was conducted using Merck aluminium sheets (Silica gel 60 F_{254}) with detection by UV absorption (254 nm), by spraying with a solution of $(NH_4)_6Mo_7O_{24}\cdot 4H_2O$ (25 g/L) and $(NH_4)_4Ce(SO_4)_4\cdot 2H_2O$ (10 g/L) in 10% sulfuric acid, a solution of $KMnO_4$ (20 g/L) and K_2CO_3 (10 g/L) in water or ninhydrin (0.75 g/L), followed by charring at ~150 ˚C. Column chromatography was performed using Screening Device b.v. Silica Gel (particle size of 40 – 63 µm, pore diameter of 60 Å) in the indicated solvents. For reversed-phase HPLC purifications an Agilent Technologies 1200 series instrument equipped with a semiprep column (Gemini C18, 250x10 mm, 5 µm particle size, Phenomenex) was used. LC/MS analysis was performed on a Surveyor HPLC system (Thermo Finnigan) equipped with a C_{18} column (Gemini, 4.6 mm x 50 mm, 5 µm particle size, Phenomenex), coupled to a LCQ Adventage Max (Thermo Finnigan) ion-trap spectrometer (ESI+). The applied buffers were H_2O, MeCN and 1% aqueous TFA. ^{1}H NMR and ^{13}C NMR spectra were recorded on a Brüker AV-400 (400 and 101 MHz respectively) or a Bruker AV-850 (850 and 214 MHz respectively) spectrometer in the given solvent. Chemical shifts are given in ppm (δ) relative to the residual solvent peak as internal standard. Coupling constants are given in Hz. High-resolution mass spectrometry (HRMS) analysis was performed with a LTQ Orbitrap mass spectrometer (Thermo Finnigan), equipped with an electronspray ion source in positive mode (source voltage 3.5 kV, sheath gas flow 10 mL/min, capillary temperature 250 °C) with resolution R = 60000 at *m*/*z* 400 (mass range *m*/*z* = 150–2000) and dioctyl phthalate (*m*/*z* = 391.28428) as a "lock mass". The high-resolution mass spectrometer was calibrated prior to measurements with a calibration mixture (Thermo Finnigan).

2-[5-(1,3,3-Trimethyl-2,3-dihydro-1H-indol-2-ylidene)-1,3-pentadi-enyl]-3,3-dimethyl-1-(6-oxo-6-(prop-2-yn-1-ylamino)hexy)-3H-indolium (1)

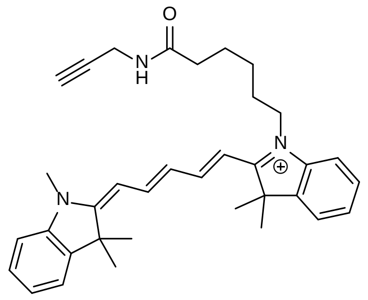

Cy5 OSu ester **1**(Kvach et al., 2008) (3.10 g, 5.0 mmol) and propargylamine (0.35 mL, 5.5 mmol) were dissolved in CH_2Cl_2 (25 mL). After addition of Et_3N (6.0 mmol, 0.84 mL), the reaction mixture was stirred for 2.5 h at room temperature. The reaction mixture was concentrated *in vacuo* and purification by column chromatography (1% MeOH in CH_2Cl_2 → 4% MeOH in CH_2Cl_2) gave Cy5-alkyne **2** (690 mg, 1.24 mmol, 25%) as a dark-blue foam. ^{1}H NMR (400 MHz, MeOD): δ 8.26 (t, *J* = 13.1 Hz, 2H), 7.48 (d, *J* = 7.4 Hz, 2H), 7.44 – 7.35 (m, 2H), 7.29 (d, *J* = 7.9 Hz, 2H), 7.24 (t, *J* = 7.4 Hz, 2H), 6.71 (t, *J* = 12.4 Hz, 1H), 6.31 (t, *J* = 13.5 Hz, 2H), 4.12 (t, *J* = 7.4 Hz, 2H), 3.92 (d, *J* = 2.5 Hz, 2H), 3.63 (s, 3H), 2.57 (t, *J* = 2.6 Hz, 1H), 2.23 (t, *J* = 7.3 Hz, 2H), 1.84 (dt, *J* = 14.9, 6.3 Hz, 2H), 1.75 – 1.66 (m, 14H), 1.51 – 1.46 (m, 2H). ^{13}C NMR (101 MHz, MeOD): δ 175.4, 175.2, 174.5, 155.4, 144.2, 143.5, 142.6, 142.5, 129.7, 129.6, 126.8, 126.1, 123.4, 123.3, 112.0, 111.8, 104.4, 55.9, 50.5, 44.8, 36.4, 31.8, 29.4, 28.2, 28.0, 27.9, 27.3, 26.4, 26.3. HRMS: Calculated for $[C_{35}H_{42}N_3O]^+$ 520.33224, found 520.33172.

Cy5-α-aziridine (4)

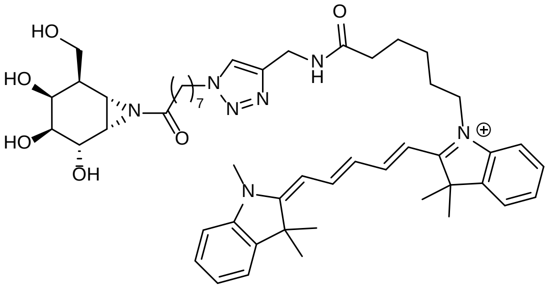

To a solution of 8-azidooctanoic acid (David et al., 2003) (94 mg, 0.50 mmol) in DMF (4 mL) was added Cy5-alkyne **2** (282 mg, 0.50 mmol) at room temperature. After addition of copper(II)sulfate pentahydrate (500 µL, 1 M in H_2O) and sodium ascorbate (700 µL, 1 M in H_2O) the reaction mixture was heated up to 80 °C and stirred overnight. The reaction mixture was concentrated *in vacuo*, redissolved in CH_2Cl_2 and washed with HCl (0.1 M, 3 x) and brine (1 x). The organic layer was dried over $MgSO_4$ and concentrated *in vacuo* to obtain Cy5 acid **3** (0.43 mmol, 0.32 g, 86%) which was used without further purification. LC/MS analysis: R_t 7.27 min linear gradient 10% -> 90% B in 12.5 min, m/z 705.33 $[M]^+$. HRMS: calculated for $[C_{43}H_{57}N_6O_3]^+$ 705.44867, found 705.44892. To a solution of crude Cy5 acid **3** (111 mg, 0.12 mmol) in DMF (0.8 mL) was added EEDQ (30 mg, 0.12 mmol) and stirred for 2 h at room temperature to obtain a pre-activated mixed anhydride solution. Aziridine **5** (17.4 mg, 0.1 mmol) was dissolved in DMF (0.4 mL) and pre-activated mixed anhydride solution (0.5 eq.) was added at 0 °C. After stirring for 30 min additional pre-activated mixed anhydride solution (0.5 eq.) was added. The reaction mixture was stirred for 30 min after which additional EEDQ (25 mg, 0.10 mmol) was added. After stirring overnight at 4 °C, the reaction mixture was quenched with MeOH at 0 °C and concentrated *in vacuo*. Purification by semi preparative reversed phase HPLC at neutral conditions (A: 50 mM NH_4HCO_3 in H_2O, B: MeCN; linear gradient: 44% → 50% B in 12 min) gave compound **4** (4.39 mg, 4.88 µmol, 5%) as a blue powder. LC/MS analysis: R_t 6.13 min and 6.37 min, linear gradient 10% -> 90% B in 12.5 min, m/z 862.40 $[M]^+$. ^{1}H NMR (850 MHz, MeOD): δ 8.24 (td, *J* = 13.2, 3.5 Hz, 2H), 7.84 (d, *J* = 4.7 Hz, 1H),

7.49 (d, J = 7.3 Hz, 2H), 7.44 – 7.39 (m, 2H), 7.32 – 7.24 (m, 4H), 6.62 (t, J = 12.3 Hz, 1H), 6.30 – 6.25 (m, 2H), 4.41 (d, J = 7.2 Hz, 2H), 4.38 – 4.34 (m, 2H), 4.11 – 4.06 (m, 3H), 3.87 – 3.84 (m, 1H), 3.63 (s, 3H), 3.59 – 3.55 (m, 2H), 3.53 (dd, J = 10.7, 6.3 Hz, 1H), 3.44 (dd, J = 10.7, 6.0 Hz, 1H), 3.36 (dd, J = 8.9, 1.9 Hz, 1H), 2.95 (dd, J = 6.0, 4.0 Hz, 1H), 2.60 (d, J = 6.0 Hz, 1H), 2.25 (t, J = 7.3 Hz, 2H), 2.01 (td, J = 7.4, 3.8 Hz, 1H), 1.88 – 1.86 (m, 2H), 1.84 – 1.79 (m, 2H), 1.74 – 1.71 (m, 13H), 1.62 – 1.55 (m, 2H), 1.48 – 1.45 (m, 2H), 1.36 – 1.27 (m, 6H). ^{13}C NMR (214 MHz, MeOD): δ 187.2, 174.3, 174.0, 173.2, 154.1, 154.0, 144.7, 142.8, 142.1, 141.2, 141.1, 128.3, 128.3, 125.2, 124.9, 124.8, 122.7, 122.0, 121.8, 110.6, 110.4, 103.0, 102.8, 72.9, 71.7, 68.1, 64.2, 61.4, 61.4, 49.7, 43.3, 43.1, 41.8, 38.0, 35.0, 34.2, 29.7, 28.7, 28.5, 28.2, 26.7, 26.5, 26.4, 25.9, 25.8, 24.9. HRMS: calculated for $[C_{50}H_{68}N_7O_6]^+$ 863.53038, found 863.52566.

The synthetic strategy towards α-galactopyranose-configured cyclophellitol aziridine ABP **4** is depicted in Scheme 1. It was envisioned that coupling of Cy5-functionalized spacer **3** with reported aziridine **5** (Willems et al., 2014) using 2-ethoxy-1-ethoxycarbonyl-1,2-dihydroquinoline (EEDQ) as the coupling reagent, would result in ABP **4**. Hence, Cy5-functionalized spacer **3** was synthesized in 2 steps from Cy5-OSu ester **1** (Kvach et al., 2008). Propargylamine was reacted with Cy5-OSu ester **1** to obtain compound **2** with an alkyne moiety. Copper(I)-catalyzed click reaction of **2** with 8-azidooctanoic acid gave Cy5-functionalized spacer **3**, which in turn was coupled to aziridine **5** to obtain ABP **4**.

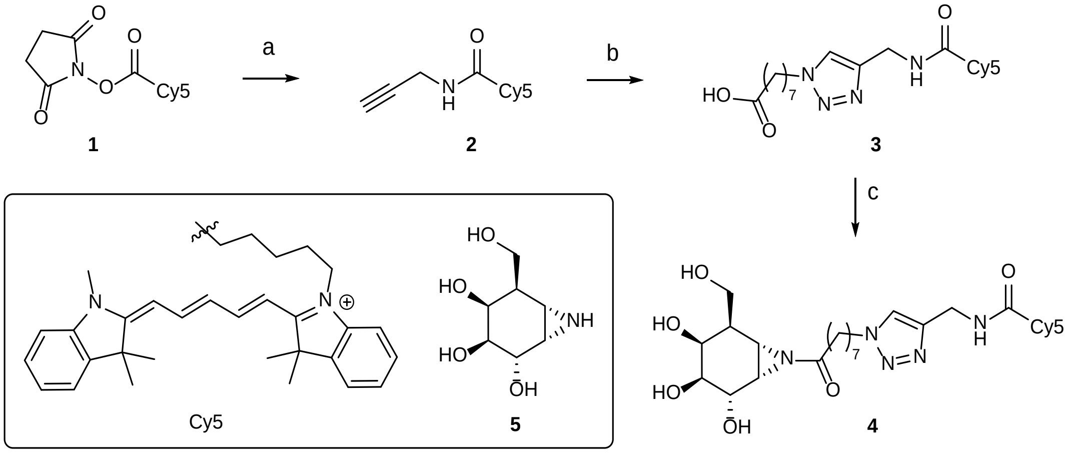

Scheme 1. Overview of synthesis. Reagent and conditions: a) propargylamine, Et_3N, CH_2Cl_2, 2.5 h, room temperature, 25%; b) 8-azidooctanoic acid, $CuSO_4 \cdot 5H_2O$, sodium ascorbate, DMF, 80 °C, overnight; c) **5** (Willems et al., 2014)**,**EEDQ, 0 °C to 4 °C, overnight, DMF, 5% over 2 steps.

(A)

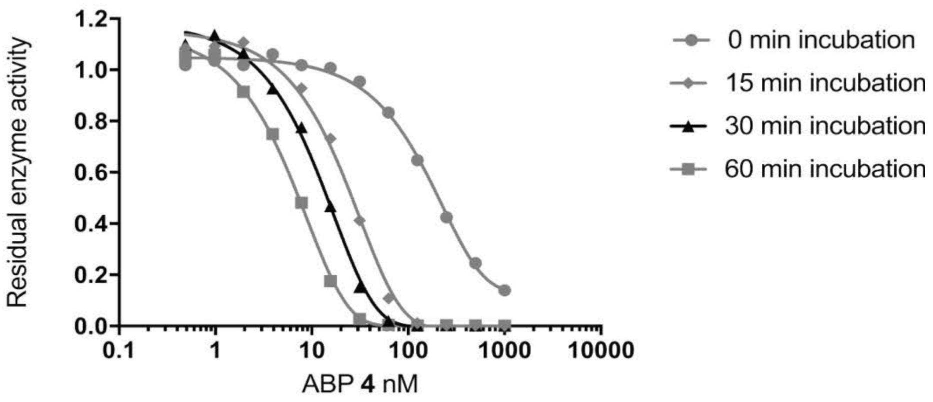

(B)

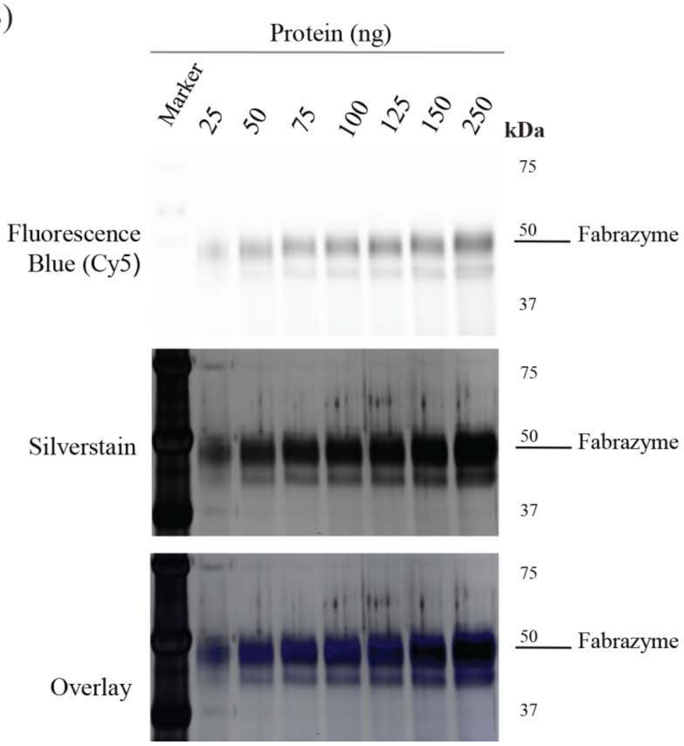

Figure S1. Characterization of ABP. (A) Inhibition of 4MU-α-GAL activity of Fabrazyme, after incubation with different ABP concentrations for different time periods. **(B)** In vitro labeling of human recombinant α-GAL, Fabrazyme.

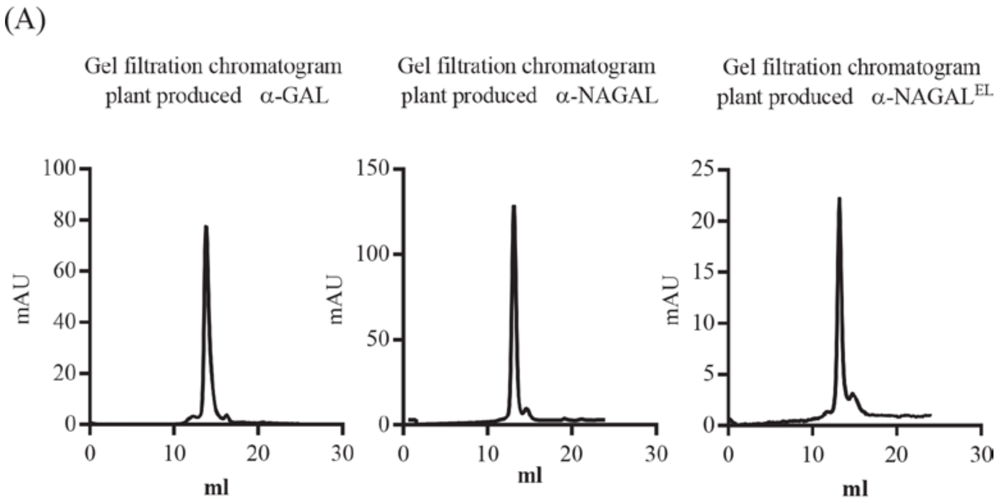

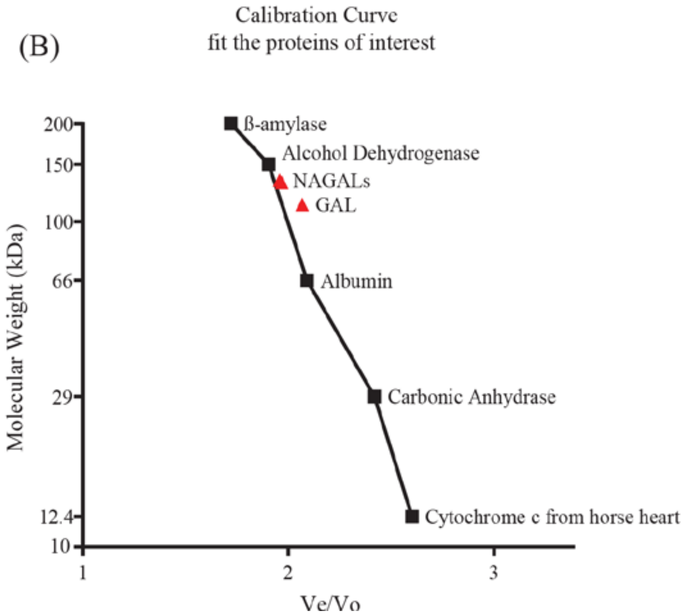

Figure S2. Gel filtration chromatograms of plant produced enzymes and calibration curve. (A) Chromatograms of all recombinant α-galactosidases after their application in Superdex™ 200 Increase 10/300 GL. **(B)** Gel filtration chromatography calibration curve of proteins with known molecular weight, fitting the results of the plant produced enzymes. Proteins used for the calibration curve were β-amylase, alcohol dehydrogenase, albumin, carbonic anhydrase, cytochrome c from horse heart and Dextran.

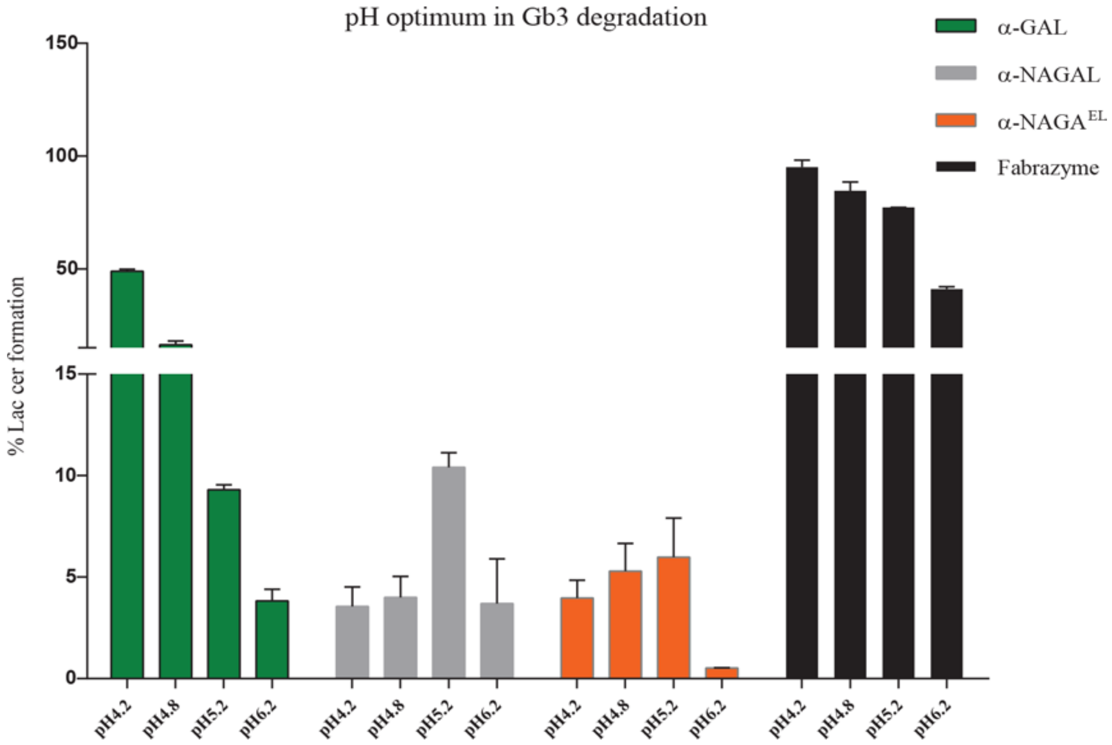

Figure S3. pH optimum of recombinant galactosidases towards Gb3. Different amounts of recombinant proteins, α-NAGAL^{EL}, α-NAGAL, α-GAL and Fabrazyme were incubated with 100pmol of C18:Gb3 overnight at 37 ^{o}C. For detection of Lac Cer formation, lipids extracted and subjected to LC-MS/MS.

References

David, O., Meester, W.J., Bieräugel, H., Schoemaker, H.E., Hiemstra, H. and van Maarseveen, J.H. (2003) Intramolecular Staudinger Ligation: A Powerful Ring-Closure Method To Form Medium-Sized Lactams. *Angewandte Chemie International Edition* **42**, 4373-4375.

Kvach, M.V., Ustinov, A.V., Stepanova, I.A., Malakhov, A.D., Skorobogatyi, M.V., Shmanai, V.V. and Korshun, V.A. (2008) A Convenient Synthesis of Cyanine Dyes: Reagents for the Labeling of Biomolecules. *European Journal of Organic Chemistry* **2008**, 2107-2117.

Willems, L.I., Beenakker, T.J.M., Murray, B., Gagestein, B., van den Elst, H., van Rijssel, E.R., Codée, J.D.C., Kallemeijn, W.W., Aerts, J.M.F.G., van der Marel, G.A. and Overkleeft, H.S. (2014) Synthesis of α- and β-Galactopyranose-Configured Isomers of Cyclophellitol and Cyclophellitol Aziridine. *European Journal of Organic Chemistry* **2014**, 6044-6056.
